# Novel Therapeutic Effects of *Euphorbia heterophylla* L. Methanol Extracts in Macular Degeneration Caused by Blue Light in A2E-Laden ARPE-19 Cells and Retina of BALB/c Mice

**DOI:** 10.3390/ph17091193

**Published:** 2024-09-10

**Authors:** Ayun Seol, Ji-Eun Kim, You-Jeong Jin, Hee-Jin Song, Yu-Jeong Roh, Tae-Ryeol Kim, Eun-Seo Park, Ki-Ho Park, So-Hae Park, Muhammad Salah Uddin, Sang-Woo Lee, Young-Woo Choi, Dae-Youn Hwang

**Affiliations:** 1 Department of Biomaterials Science (BK21 FOUR Program), Life and Industry Convergence Research Institute, College of Natural Resources and Life Science, Pusan National University, Miryang 50463, Republic of Korea; a990609@naver.com (A.S.); prettyjiunx@naver.com (J.-E.K.); hjinyuu1@naver.com (Y.-J.J.); hejin1544@naver.com (H.-J.S.); buzyu99@naver.com (Y.-J.R.); xofuf0701@naver.com (T.-R.K.); geg9393@naver.com (E.-S.P.); pujihao@naver.com (K.-H.P.); sohaehw@pusan.ac.kr (S.-H.P.); ychoi@pusan.ac.kr (Y.-W.C.); 2 Ethnobotanical Database of Bangladesh, Dacca 1208, Bangladesh; plantsofbd@gmail.com; 3 International Biological Material Research Center, Korea Research Institute of Bioscience and Biotechnology, Daejeon 34141, Republic of Korea; ethnolee@kribb.re.kr; 4 Longevity & Wellbeing Research Center, Laboratory Animals Resources Center, Pusan National University, Miryang 50463, Republic of Korea

**Keywords:** *Euphorbia heterophylla* L., age-related macular degeneration, retina, oxidative stress, BALB/c mice

## Abstract

Natural products with high antioxidant activity are considered as innovative prevention strategies to effectively prevent age-related macular degeneration (AMD) in the early stage because the generation of reactive oxygen species (ROS) leading to the development of drusen is reported as an important cause of this disease. To investigate the prevention effects of the methanol extracts of *Euphorbia heterophylla* L. (MEE) on AMD, its effects on the antioxidant activity, inflammatory response, apoptosis pathway, neovascularization, and retinal tissue degeneration were analyzed in N-retinylidene-N-retinylethanolamine (A2E)-landed spontaneously arising retinal pigment epithelia (ARPE)-19 cells and BALB/c mice after exposure to blue light (BL). The MEE contained 10 active components and showed high free radical scavenging activity against 2,2-diphenyl-1-picrylhydrazyl (DPPH), 2,2′-azino-bis (3-ethylbenzothiazoline-6-sulfonic acid) (ABTS), and nitric oxide (NO) radicals. The pretreatments of high-dose MEE remarkably suppressed the production of intracellular ROS (88.2%) and NO (25.2%) and enhanced (SOD) activity (84%) and the phosphorylation of nuclear factor erythroid 2–related factor 2 (Nrf2) in A2E + BL-treated ARPE-19 cells compared to Vehicle-treated group. The activation of the inducible nitric oxide synthase (iNOS)-induced cyclooxygenase-2 (COX-2) mediated pathway, inflammasome activation, and expression of inflammatory cytokines was significantly inhibited in A2E + BL-treated ARPE-19 cells after the MEE pretreatment. The activation of the apoptosis pathway and increased expression of neovascular proteins (36% for matrix metalloproteinase (MMP)-9) were inhibited in the MEE pretreated groups compared to the Vehicle-treated group. Furthermore, the thickness of the whole retina (31%), outer nuclear layer (ONL), inner nuclear layer (INL), and photoreceptor layer (PL) were significantly increased by the MEE pretreatment of BALB/c mice with BL-induced retinal degeneration. Therefore, these results suggest that the MEE, with its high antioxidative activity, protects against BL-induced retinal degeneration through the regulation of the antioxidative system, inflammatory response, apoptosis, and neovascularization in the AMD mouse model.

## 1. Introduction

Age-related macular degeneration (AMD) is an important cause of the severe loss of central vision in older adults. AMD is characterized by the deposition of drusen and retinal pigment epithelium (RPE) abnormalities (non-neovascular derangement, dry AMD) and choroidal neovascular membrane (CNV) formation (neovascular derangement, wet AMD) [1,2]. Dry AMD accounts for more than 90% of the total AMD patients and usually progresses slowly over several years with aging [3]. In normal conditions, retinol, which is a vitamin A metabolite and an important compound in the visual cycle, polymerizes to 11-cis-retinol and transforms to all-trans-retinal accumulated in the photoreceptor [4,5]. This molecule with high reactivity is a major constituent to the formation of N-retinylidene-N-retinylethanolamine (A2E) through the condensation of all-trans-retinal-PE adduct fraction [6]. Then, A2E and other fluorescent chromophores are transported to RPE and breakdown by the process of lysosomal degradation [7]. However, in advanced age, they are accumulated as the form of granule called lipofuscin in RPE [6]. When lipofuscin is exposed to visible light, A2E in drusen forms an epoxide structure as potential photosensitizers [8]. The A2E-epoxide generates large amounts of reactive oxygen species (ROS) and several byproducts [9,10,11]. The overproduction of these radicals induces the dysfunction of RPE cells through damage to the anti-oxidative system, mitochondrial dysfunction, extracellular matrix (ECM) dysregulation, and accumulation of lipidic and protein aggregation [12,13,14,15]. In experiments involving cellular conditions, the pathological features of AMD in retinal cells are induced using a combination of two key factors—A2E and blue light (BL)—that are associated with the molecular mechanisms of this disease. Most previous studies show that AMD is successfully induced in A2E-treated RPE cells after the expose of BL with a wavelength of 430 nm [16,17]. In addition, similar pathological conditions were observed in light-induced AMD animal models that were exposed to intense or prolonged light. Significant retinal damage is chronic and progresses slowly over time, although it can occur selectively in certain retinal cell types, such as photoreceptors [18]. However, this model differs from the whole retina acute photodamage models that show acute macular neuroretinopathy, retinal detachment, and retinal bleeding, as well as wide and clear retinal damage patterns rapidly induced by phototoxic substances or high-intensity light [19]. Therefore, light-induced AMD animal models are more suitable for evaluating therapeutic effects, as they can reproduce the main features of human AMD.

Meanwhile, few standard therapeutic strategies have been proposed to slow the progression of AMD in its final stages, and no ideal preventive treatment is currently available [20]. Anti-vascular endothelial growth factor (VEGF) agents for limiting the function of VEGF are considered as recommended standard treatments for wet AMD, while several intravitreal complement inhibitors that target complement component 3 (C3) and complement component 5 (C5) offer a potential therapeutic option for dry AMD [21,22]. Also, the natural drugs and products with high antioxidant activity have been reported as one of prevention treatment strategies for AMD patients. The prevention of AMD progression and the significant improvement in vision acuity have been observed with a treatment of lutein, β-carotene, eicosapentaenoic acid (EPA), vitamin C, and omega-3 [21,23,24,25,26]. Especially, the remarkable therapeutic effects are detected in several natural products including *Aronia melanocarpa* [27], bilberry [28], *Solanum melongena* L. [29], *Spirulina maxima* [30], and *Dipterocarpus tuberculatus* [31]. As part of the above research, novel natural products with significant therapeutic efficacy in treating macular degeneration have received a lot of attention as therapeutic drug candidates. We focused on *Euphorbia heterophylla* L. among various herbal medicines. This plant is a species of the *Euphorbiaceae* family that has long been used to treat various health problems including skin diseases, warts, migraines, gonorrhea, and parasitic infections [32]. It is widely distributed as annual flowering plant in tropical and subtropical America and in South and Southeast Asia [33]. There have been limited investigations into the medicinal efficacy and mechanism of action of this plant to date. Actually, the essential oil and the reduced graphene oxide (rGO) of *E. heterophylla* L. exhibit high scavenging activity against free radicals and anti-cancer effects [34,35]. Furthermore, their ethyl acetate and methanol extracts showed a significantly high Fe^3+^ and Cu^2+^ reducing potential and total antioxidant capacity [36]. However, no scientific evidence has been provided for the protective effects and the mechanism of action of *E. heterophylla* L. in AMD.

In this study, the protective effects of the methanol extracts of *Euphorbia heterophylla* L. stems and leaves (MEE) were investigated in both in vitro and in vivo AMD models established by exposure to BL in A2E-laden arising retinal pigment epithelia (ARPE)-19 cells and BALB/c mice, respectively, to evaluate its potential as a treatment for AMD. Our investigation specifically focused on the mechanisms related to antioxidant activity, inflammatory response, apoptosis, and neovascularization.

## 2. Results

### 2.1. Identification of Bioactive Compounds in the MEE

**Figure 1:**
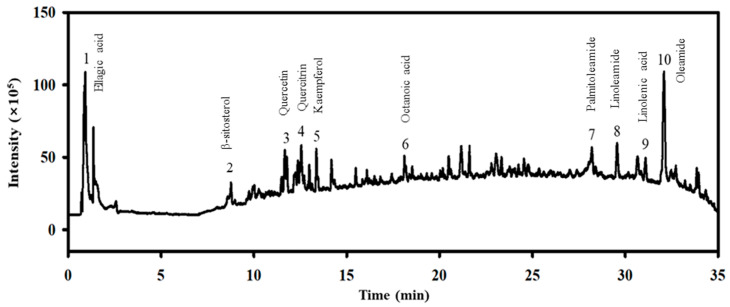
Determination of the main components in the MEE, using LC-MS analysis. Ten compounds including oleamide, linoleamide, palmitoleamide, quercetin, quercetrin, kaempferol, ellagic acid, β-sitosterol, octanoic acid, and linoleic acid were detected as distinct peaks in the chromatogram. Abbreviations: LC-MS, liquid chromatography–mass spectrometry; MEE, methanol extracts of *Euphorbia heterophylla* L.

First, we identified the bioactive compounds in the MEE to predict which compounds were associated with the therapeutic effects of the MEE in AMD treatment. The ultra-high-performance liquid chromatography–mass spectrometry (UHPLC-MS) analyses detected 10 bioactive compounds in the MEE. Among these, oleamide was present in the highest relative content, followed by linoleamide, palmitoleamide, quercetin, quercetrin, kaempferol, and ellagic acid (Figure 1 and Figure S1, Table 1). The above results suggest that the MEE has the potential for use as a therapeutic drug for AMD. This is because ellagic acid, β-sitosterol, and quercetin possess excellent antioxidant properties, with quercetin, in particular, being able to protect RPE cells from oxidative damage and cellular aging [37].

### 2.2. Antioxidative Activity of the MEE against Free Radicals

**Figure 2:**
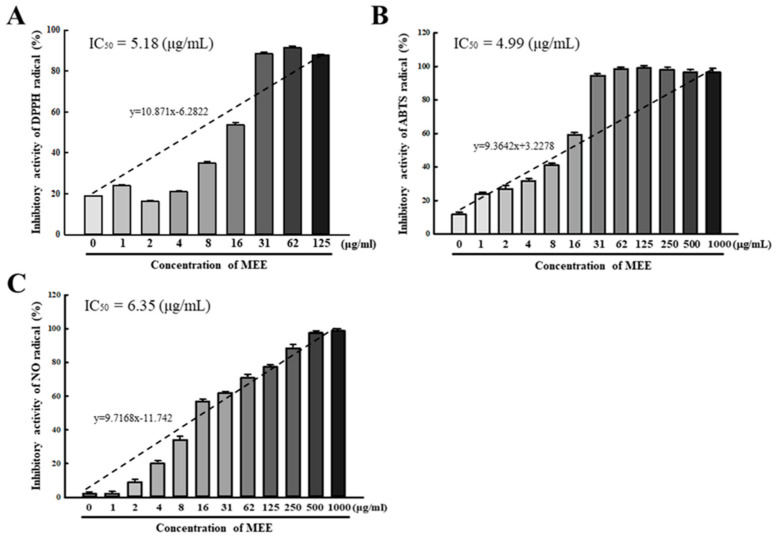
Free radical scavenging activity of the MEE. (**A**) DPPH radical scavenging activity. (**B**) ABTS radical scavenging activity. (**C**) NO radical scavenging activity. The activity of each radical was determined at 0.1 mM radicals and varying concentrations of the MEE (1–1000 μg/mL). The dotted line represented the trend pattern. The free radical scavenging analyses were performed on three MEE samples, and the optical density was measured twice for each well. Data are reported as the mean ± SD. Abbreviations: DPPH, 2,2-diphenyl-1-picrylhydrazyl; ABTS, 2,2′-azino-bis (3-ethylbenzothiazoline-6-sulfonic acid); NO, nitric oxide; IC_50_, half-maximal inhibitory concentration; MEE, methanol extracts of *Euphorbia heterophylla* L.

To predict the potential utility of the MEE as a therapeutic drug for AMD, the antioxidative activity of the MEE was evaluated against three free radicals, namely, 2,2-diphenyl-1-picrylhydrazyl (DPPH), 2,2′-azino-bis (3-ethylbenzothiazoline-6-sulfonic acid) (ABTS), and nitric oxide (NO). The MEE showed high inhibitory activity against these three free radicals in a significant dose-dependent manner. These levels were remarkably increased at 1–1000 µg/mL of the MEE, and the half-maximal inhibitory concentration (IC_50_) value for DPPH, ABTS, and NO radicals was determined to be 5.18 µg/mL, 4.99 µg/mL, and 6.35 µg/mL, respectively, (Figure 2A–C). These results suggest that the MEE may have strong antioxidative activity and could prevent the progression of the disease.

### 2.3. Suppression of Oxidative Stress by the MEE in A2E + BL-Exposed ARPE-19 Cells

**Figure 3:**
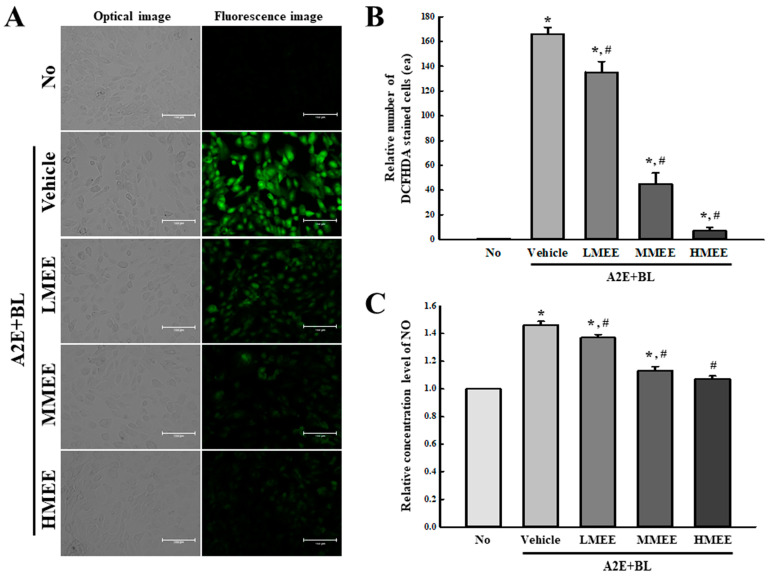
Detection of intracellular ROS and NO in MEE + A2E + BL-treated ARPE-19 cells. (**A**) Fluorescence image of DCF stained ARPE-19 cells. The fluorescence of the cells was observed at 200× magnification. (**B**) Number of DCF stained cells. The preparation of DCFH-DA-stained cells was performed on two to three wells per group, and the positive cells were counted in two fields of view (67,500 mm^2^) in each well. (**C**) NO concentration. NO concentration in the culture supernatants was measured using the Griess reagent. The NO concentration analyses were performed using three wells per each group, and the assay for each sample was analyzed twice. Data are reported as the mean ± SD. * *p* < 0.05 vs. No group. # *p* < 0.05 vs. Vehicle + A2E + BL-treated group. Abbreviations: NO, nitric oxide; DCFHDA, 2,7-Dichlorofluorescin diacetate; MEE, methanol extracts of *Euphorbia heterophylla* L.; A2E, N-retinylidene-N-retinylethanolamine; BL, blue light.

To examine whether the MEE pretreatment could suppress the oxidative stress that leads to AMD, the alterations in the production of intracellular ROS and NO were measured in A2E + BL-exposed ARPE-19 cells after the MEE pretreatment. A significant dose-dependent decrease in the number of dichlorofluorescein (DCF)-stained cells representing intracellular ROS was detected in the three MEE + A2E + BL-treated groups when compared with the Vehicle + A2E + BL-treated group. The highest decrease at 88.2% was observed in the HMEE + A2E + BL-treated group (Figure 3A,B). Also, a similar pattern on the significant dose-dependent decrease was observed in the intracellular NO in the same groups, although the decrease rate in the number of DCF-stained cells was smaller (Figure 3C). Three MEE + A2E + BL-treated groups showed a decrease of 6.1%, 21.8%, and 25.2%. Thus, these results indicate that the MEE pretreatment can suppress intracellular ROS and NO production in A2E + BL-treated ARPE-19 cells and may contribute to the decrease in oxidative stress in the AMD model.

### 2.4. Antioxidant Activity of the MEE in A2E + BL-Exposed ARPE-19 Cells

**Figure 4:**
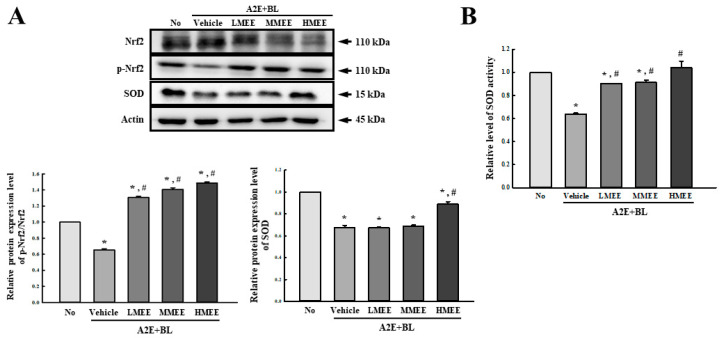
Determination of SOD, Nrf2, and p-Nrf2 proteins levels and SOD activity in MEE + A2E + BL-treated ARPE-19 cells. (**A**) Expression level of SOD, Nrf2, and p-Nrf2 proteins. After preparation of the total cell lysates from MEE + A2E + BL-treated ARPE-19 cells, SOD, Nrf2, and p-Nrf2 proteins were analyzed using specific antibodies. The expression level of each protein was normalized to β-actin. The cell lysates were prepared from two to three dishes per group and the Western blots were analyzed twice for each sample. (**B**) Level of SOD activity. After preparation of total cell lysates from the MEE + A2E + BL-treated ARPE-19 cells, SOD activity was detected using a specific assay kit. One unit of SOD was defined as the amount of the enzyme in the MEE solution (20 µL) that inhibits the reduction reaction of WST-1 with the superoxide anion by 50%. Data are reported as the mean ± SD. * *p* < 0.05 vs. No group. # *p* < 0.05 vs. Vehicle + A2E + BL-treated group. Abbreviations: SOD, superoxide dismutase; Nrf2, nuclear factors erythroid 2-related factors; MEE, methanol extracts of *Euphorbia heterophylla* L.; A2E, N-retinylidene-N-retinylethanolamine; BL, blue light; WST-1, water-soluble tetrazolium salt-1; ARPE, arising retinal pigment epithelia.

To investigate whether the suppressive effects of MEE on oxidative stress accompany an improvement in antioxidative capacity, the alterations in the phosphorylation of nuclear factors erythroid 2-related factors (Nrf2) and the activity and expression of superoxide dismutase (SOD) were examined in A2E + BL-exposed ARPE-19 cells after the MEE pretreatment. The level of phosphorylation of Nrf2 was lower in the Vehicle + A2E + BL-treated group than in the control group. However, this level was significantly increased with dose-dependent pattern in the three MEE + A2E + BL-treated groups compared to the Vehicle + A2E + BL-treated group (Figure 4A). Also, the expression level of the SOD protein was increased in the MEE + A2E + BL-treated group, although a significant increase was detected only in the HMEE + A2E + BL-treated group (Figure 4A). Furthermore, the expression pattern of the SOD protein was successfully reflected in the relative activity of the SOD. These levels were remarkably enhanced in the three MEE + A2E + BL-treated groups, although there were some differences in the LMEE + A2E + BL and MMEE + A2E + BL-treated groups (Figure 4B). Therefore, these results suggest that the suppressive effects of the MEE on oxidative stress may be tightly associated with the improvement in antioxidative capacity, which was reduced due to A2E + BL exposure.

### 2.5. Suppression of Inflammatory Response in A2E + BL-Exposed ARPE-19 Cells by the MEE

**Figure 5:**
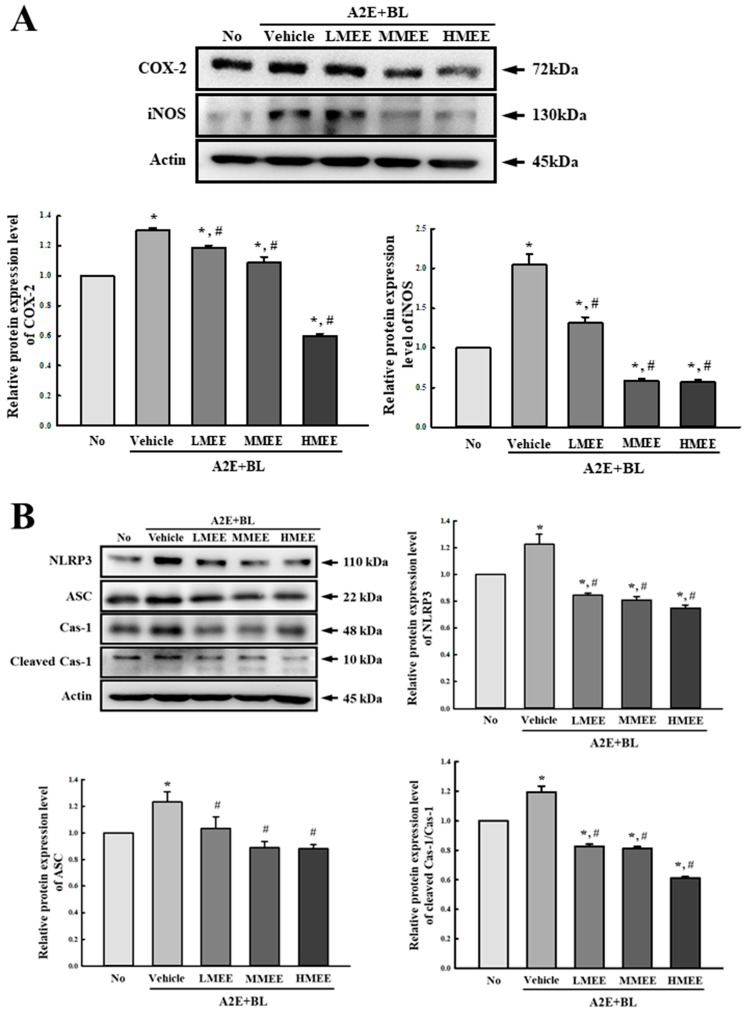

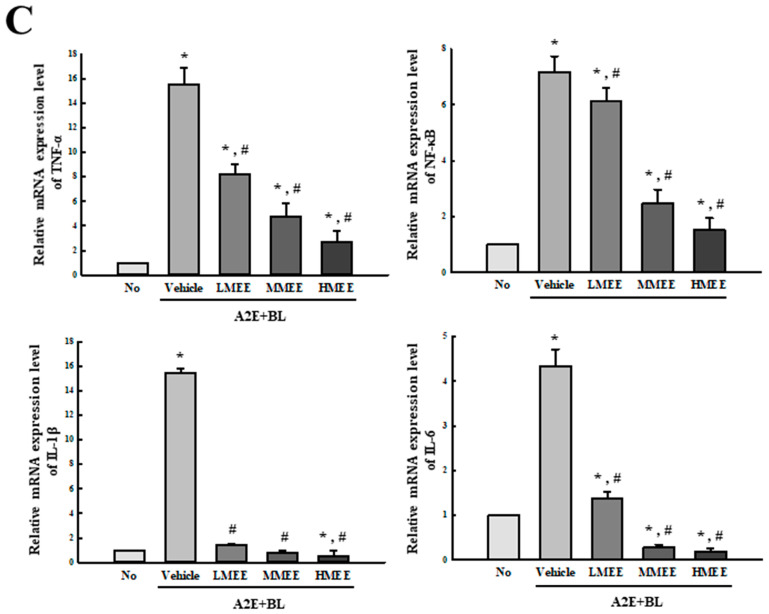
Expression of key regulators on the iNOS-induced COX-2 mediated pathway, inflammasome pathway and transcription of inflammatory cytokines in MEE + A2E + BL-treated ARPE-19 cells. (**A**) Expression level of COX-2 and iNOS. (**B**) Expression level of NLRP3, ASC, Cas-1, and Cleaved Cas-1. After preparation of the total cell lysates from MEE + A2E + BL-treated ARPE-19 cells, the expression level of each protein was analyzed using specific antibodies and normalized to β-actin. The cell lysates were prepared from two to three dishes per group and the Western blots were analyzed twice for each sample. (**C**) mRNA levels of TNF-α, IL-1β, IL-6, and NF-κB. The mRNA levels of each gene were analyzed using specific primers and normalized to β-actin. The total RNAs were purified from cells of two to three dishes per group and RT-qPCR analysis was conducted twice for each sample. Data are reported as the mean ± SD. * *p* < 0.05 vs. No group. # *p* < 0.05 vs. Vehicle + A2E + BL-treated group. Abbreviations: COX-2, cyclooxygenase-2; iNOS, inducible nitric oxide synthase; NLRP3, NLR family pyrin domain containing 3; ASC, apoptosis-associated speck-like protein; Cas-1, Caspase-1; TNF-α, tumor necrosis factor α; IL, Interleukin; NF-κB, nuclear factor kappa-light-chain-enhancer of activated B; MEE, methanol extracts of *Euphorbia heterophylla* L.; A2E, N-retinylidene-N-retinylethanolamine; BL, blue light; ARPE, arising retinal pigment epithelia.

To investigate the effects of the MEE in lowering the inflammatory responses caused by the A2E + BL exposure, alterations in the inducible nitric oxide synthase (iNOS)-induced cyclooxygenase-2 (COX-2) mediated pathway, inflammasome pathway, and the expression of inflammatory cytokines were analyzed in the A2E + BL-exposed ARPE-19 cells after the MEE pretreatment. The expression levels of the iNOS and COX-2 was significantly enhanced in the Vehicle + A2E + BL-treated group compared to the No group. However, these levels decreased in a dose-dependent manner in the three MEE-treated groups, although the decrease in the expression of COX-2 protein was greater than that of the iNOS protein (Figure 5A). In addition, the suppression of two proteins expression by the MEE was accompanied by the inhibition of inflammasome activation. The expressions of the NLR family pyrin domain containing 3 (NLRP3), apoptosis-associated speck-like protein (ASC), and the Cleaved Caspase (Cas)-1/Cas-1 proteins were increased after the exposure to A2E + BL. However, the levels of these three proteins were remarkably decreased in the LMEE-, MMEE-, and HMEE + A2E + BL-treated groups compared to the control group. Specifically, the decrease in the Cleaved Cas-1/Cas-1 proteins was the highest in the MEE + A2E + BL-treated group (Figure 5B). Furthermore, the transcription level of inflammatory cytokines was successfully reflected in the suppressive effects of the MEE on the regulation of the iNOS-induced COX-2 mediated pathway and the inflammasome pathway. The levels of tumor necrosis factor α (TNF-α), nuclear factor kappa-light-chain-enhancer of activated B (NF-κB), and interleukin (IL)-1β and IL-6 mRNA were remarkably higher in the Vehicle + A2E + BL-treated group than the control group. But, they were significantly decreased with a dose-dependent manner in the three MEE + A2E + BL-treated groups when compared to the Vehicle + A2E + BL-treated group (Figure 5C). Taken together, above results show that the MEE pretreatment can suppress the inflammatory response caused by A2E + BL exposure in ARPE-19 cells through the regulation of the iNOS-induced COX-2 mediated pathway and the NLRP3 inflammasome pathway.

### 2.6. Suppression of Cell Death by the MEE in A2E + BL-Exposed ARPE-19 Cells

**Figure 6:**
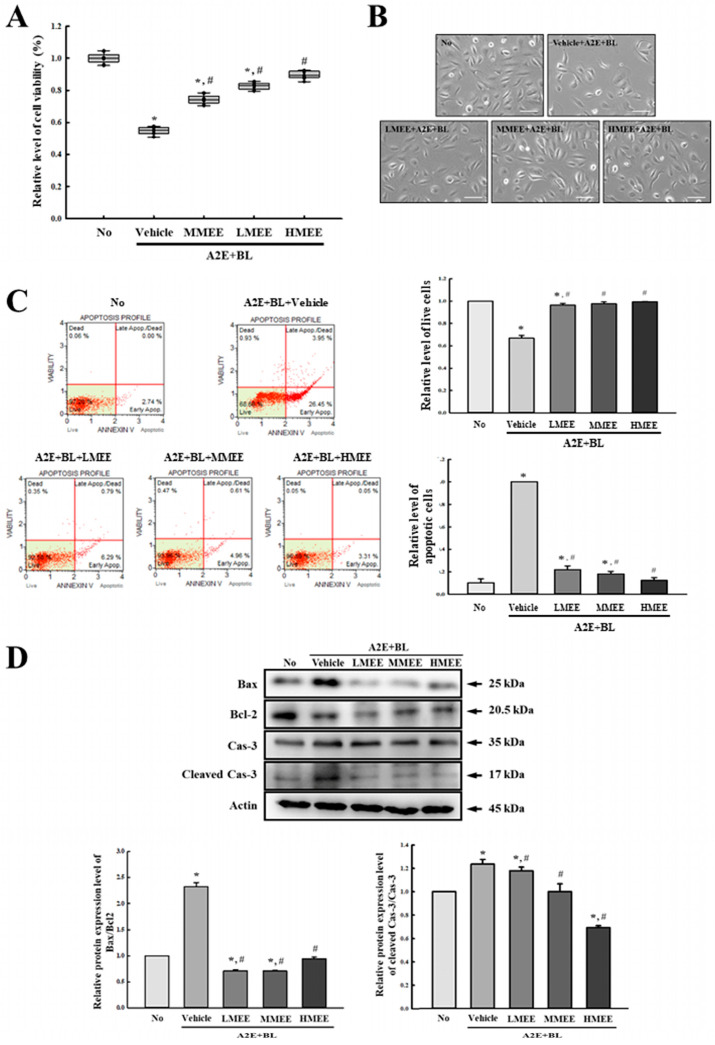
Level of cell deaths parameters in the MEE + A2E + BL-treated ARPE-19 cells. (**A**) Relative level of cell viability and (**B**) morphology of the MEE + A2E + BL-treated ARPE-19 cells. After pretreatment with three different dosages of the MEE for 24 h, their viability was analyzed using the MTT assay. The MTT assays were performed on samples from two to three wells per group, and the optical density was measured twice for each well. (**C**) The number of specific cells stained with Annexin V and 7-AAD. After staining, the number of stained cells was analyzed using a Muse Cell Analyzer. Annexin V and 7-AAD staining were performed from two to three wells per group, and the number of specific cells was counted twice for each well. The red lines represented four different stages of cell death, while individual cell was marked with a red dot. (**D**) Protein expression level of Bax, Bcl-2, Cas-3, and Cleaved Cas-3. The expression level of each protein was normalized to β-actin. The cell lysates were prepared from two to three dishes per group and the Western blots were analyzed twice for each sample. Data are reported as the mean ± SD. * *p* < 0.05 vs. No group. # *p* < 0.05 vs. Vehicle + A2E + BL-treated group. Abbreviations: MTT, 3-(4,5-dimethylthiazol-2-yl)-2,5-diphenyltetrazolium bromide; 7-AAD, 7-aminoactinomycin D; Bax, Bcl-2-associated X protein; Bcl-2, B-cell lymphoma 2; Cas-3, Caspase-3; MEE, methanol extracts of *Euphorbia heterophylla* L.; A2E, N-retinylidene-N-retinylethanolamine; BL, blue light; ARPE, arising retinal pigment epithelia.

To investigate the suppressive effects of MEE on the cell death caused by the A2E + BL exposure, the alterations in cell viability, apoptotic cell population, and expression of apoptotic proteins were analyzed in the A2E + BL-exposed ARPE-19 cells after the MEE pretreatment. The cell viability was significantly enhanced in the three MEE + A2E + BL-treated groups although these levels were remarkably lower in the Vehicle + A2E + BL-treated group than in the control group. Also, the morphology of the ARPE-19 cells was well reflected at the levels of cell viability (Figure 6A,B). In addition, the population of apoptotic and live cells was analyzed in the three MEE + A2E + BL-treated groups after staining with the Annexin V/propidium iodide (PI) detection kit to examine whether the effects of the MEE on A2E + BL-induced cell death are associated with the mechanism of apoptosis. The number of live cells was decreased by 35% in the Vehicle + A2E + BL-treated group compared to the No group. However, these levels were completely recovered to the No group after the MEE pretreatment. Furthermore, a reverse pattern was observed in the number of apoptotic cells. The number of apoptotic cells significantly decreased by 79%, 81%, and 87% in the LMEE-, MMEE-, and HMEE + A2E + BL-treated groups, respectively, compared with the No group. They showed a significant dose-dependent manner and the best treatment effects were detected in HMEE + A2E + BL-treated group (Figure 6C and Table 2). Moreover, the effects of the MEE on the decreased number of apoptotic cells were successfully reflected in the expression of the regulatory proteins for apoptosis. The increased levels of the Bcl-2-associated X protein (Bax)/B-cell lymphoma 2 (Bcl-2) ratio and the Cleaved Cas-3/Cas-3 ratio caused by the A2E + BL exposure were remarkably decreased in the three MEE + A2E + BL-treated groups. Specifically, a dose-dependent decrease was detected in the protein expression level of Cleaved Cas-3/Cas-3 (Figure 6D). Therefore, these results indicate that the MEE pretreatment can suppress the cell death caused by A2E + BL exposure through the regulation of apoptosis.

### 2.7. Improvement in the Regulation of Angiogenesis by the MEE in A2E + BL-Exposed ARPE-19 Cells

**Figure 7:**
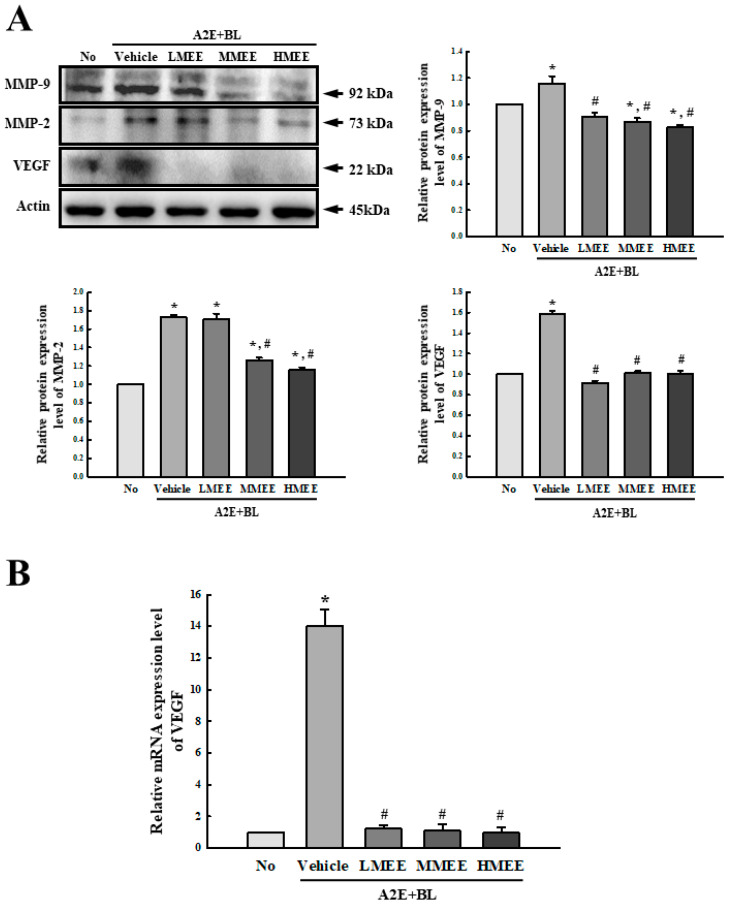
Expression levels of angiogenic factors in MEE + A2E + BL-treated ARPE-19 cells. (**A**) Expression of angiogenic proteins. After collections of the total cell lysates from the MEE + A2E + BL-treated ARPE-19 cells, the MMP-2, MMP-9, and VEGF proteins were analyzed using specific antibodies. The expression level of each protein was normalized to β-actin. The cell lysates were prepared from two to three dishes per group and the Western blots were analyzed twice for each sample. (**B**) Transcription level of VEGF gene. The mRNA levels of this gene were analyzed using specific primers and normalized to β-actin. The total RNAs were purified from the cells of two to three dishes per group and RT-qPCR analysis was conducted twice for each sample. Data are reported as the mean ± SD. * *p* < 0.05 vs. No group. # *p* < 0.05 vs. Vehicle + A2E + BL-treated group. Abbreviations: MMP, matrix metalloproteinase; VEGF, vascular endothelial growth factor; MEE, methanol extracts of *Euphorbia heterophylla* L.; A2E, N-retinylidene-N-retinylethanolamine; BL, blue light; ARPE, arising retinal pigment epithelia.

Next, we analyzed the expression levels of matrix metalloproteinase (MMP)-2, MMP-9 and VEGF proteins in the MEE + A2E + BL-treated ARPE-19 cells to examine the improvement in the dysregulation of angiogenesis caused by the A2E + BL exposure by the MEE. The expression levels of the three proteins, including MMP-2, MMP-9, and VEGF, were higher in the Vehicle + A2E + BL-treated group than in the control group. But, these expression levels were significantly declined after the MEE pretreatment at different doses. Especially, only MMP-9 proteins showed a dose-dependent response in the three MEE + A2E + BL-treated groups (Figure 7). The above results indicate that the MEE pretreatment may induce the downregulation of angiogenic proteins during the dysregulation of angiogenesis caused by the A2E + BL exposure.

### 2.8. Verification of the In Vitro Effects of the MEE on the BL-Induced Photoreceptor Degranulation in the Retina of BALB/c Mice

**Figure 8:**
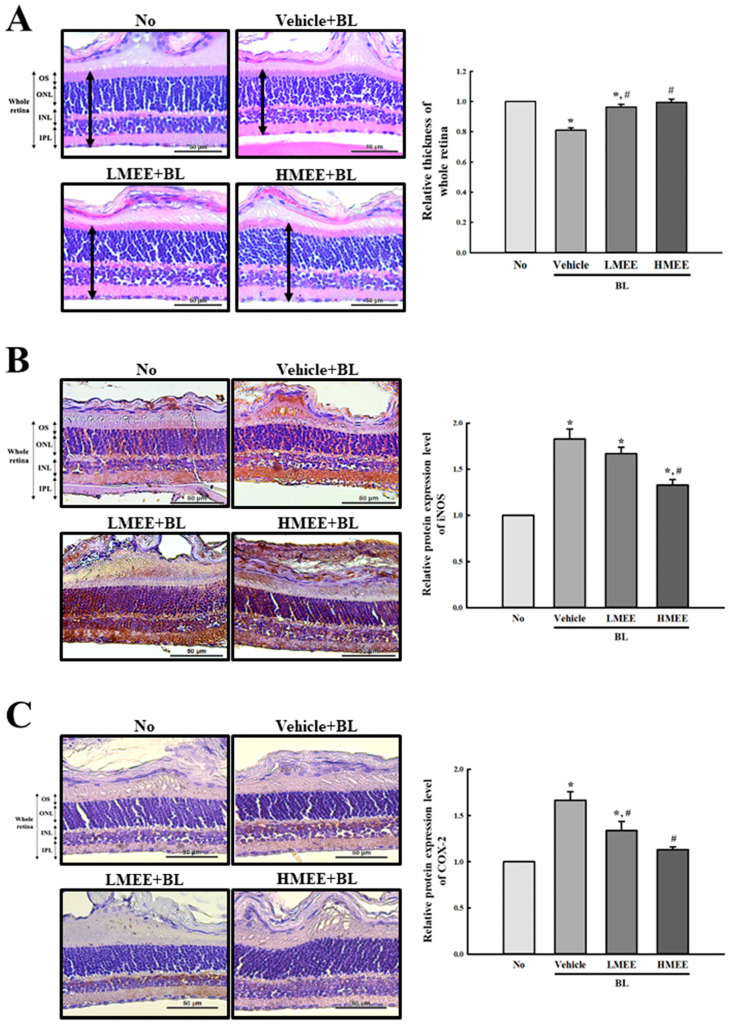
Histological structures in the retina and expression of iNOS and COX-2 proteins of MEE + BL-treated BALB/c mice. (**A**) Histology of the retina and thickness of whole retina. Histopathological alterations such as the thickness of the whole retina, OS, ONL, INL, and IPL were observed in the retina section stained with H&E solution at 200× magnification. The thickness of whole retina represented bold arrow. The thicknesses of retina were measured using the Image J program 1.52a. Three different points including thickest point, thinnest point and medium thickness point were measured and presented as their mean values. The preparation of the H&E-stained section was performed on three to five mice per group and the thickness was measured twice for each stained tissue. Expression of (**B**) iNOS and (**C**) COX-2 proteins in the retina. After staining three different antibodies, the stable DAB developed retinal section was observed at 400× magnification using light microscopy. The preparation of the IHC stained section was performed on three to five mice per group, and the distribution of each protein was analyzed twice for each stained tissue. Data are reported as the mean ± SD. * *p* < 0.05 vs. No group. # *p* < 0.05 vs. Vehicle + A2E + BL-treated group. Abbreviations: OS, outer segment, ONL, outer nuclear layer; INL, inner nuclear layer; IPL, inner plexiform layer; iNOS, inducible nitric oxide synthase; COX-2, cyclooxygenase-2; MEE, methanol extracts of *Euphorbia heterophylla* L.; A2E, N-retinylidene-N-retinylethanolamine; BL, blue light; H&E, hematoxylin, and eosin; DAB, 3,3′-diaminobenzidine; IHC, immunohistochemistry.

Finally, we verified the therapeutic effects of MEE treatment seen in the A2E + BL-exposed ARPE-19 cells using an animal model. To achieve this, the alterations in the thickness of the whole retina, outer segment (OS), outer nuclear layer (ONL), inner nuclear layer (INL), and inner plexiform layer (IPL) were analyzed in BL-exposed BALB/c mice after the MEE pretreatment. The thickness of the whole retina was significantly enhanced in the LMEE + BL- and HMEE + BL-treated groups compared with the Vehicle + BL-treated group, although they were lower in the Vehicle + BL-treated group than in the non-treated group (Figure 8A). Also, a similar improvement was measured in the thickness of the OS, ONL, IPL, and INL. After the MEE pretreatment, these levels were significantly increased in the BL-exposed retinas of BALB/c mice (Table 3).

Furthermore, we investigated whether the improvement in the regulation of retina thickness seen with the MEE treatment has an association with the alteration of the inflammatory response. To achieve this, the alteration in the expression of key parameters of the iNOS and COX-2 proteins was measured in the retinal tissues of the BL-exposed BALB/c mice. As shown in Figure 8B,C, the increased density of the iNOS and COX-2 in the control group, the dark brown color was significantly decreased in a dose-dependent manner in the LMEE + BL- and HMEE + BL-treated groups, although the decrease rate varied. Therefore, these results indicate that the improvement seen in the ARPE-19 cells with MEE treatment could be successfully verified in the BL-exposed BALB/c mice model with retinal degeneration. These results suggest that the MEE, with its high anti-oxidative activity, may have great potential in the treatment of AMD.

## 3. Discussion

AMD is a complex, progressive, and multifactorial disease affecting the retinas of older people [38]. In AMD pathogenesis, the dysfunction and degeneration of RPE are induced through the ROS-mediated dysregulation of cellular control as a result of abnormal misfolded proteins and abnormal lipids accumulating to form drusen [39]. Because of the pathogenetic mechanism mediated through oxidative stress, the administration of ROS scavengers can be applied to protect against or delay the progression of early AMD [40]. Specifically, significant research has been focused on identifying natural products with appreciable antioxidant activity. Our study sought to identify novel natural products to protect against AMD and investigate the therapeutic effects and molecular mechanism of the MEE in ARPE-19 cells and BALB/c mice with AMD phenotypes. Therefore, the results of the present study suggest that the MEE may play a significant role in protecting against BL-induced macular degeneration through the inhibition of oxidative stress, inflammation, apoptosis, and neovascularization and by improving retinal thickness. However, dose–response analyses for animal models are needed to provide more robust data on the efficacy and potential toxicity of varying doses, which is critical for clinical translation.

We selected a novel candidate, *E. heterophylla* L. for investigating its properties in AMD treatment because the plant extracts exhibit high antioxidant properties. Essential oil from the aerial parts of this plant showed a significant scavenging activity for DPPH and H_2_O_2_ free radicals, and the IC_50_ values were 325 μL/L and 431.4 μL/L [34]. Also, high antioxidant activity against DPPH (194.28 ± 0.22 mg Trolox (TE)/g, 193.55 ± 0.21 mg TE/g) and ABTS (324.65 ± 0.77mg TE/g, 323.76 ± 0.56 mg TE/g) was detected in the methanol and aqueous extracts of *E. heterophylla* L. [36]. Similar antioxidant activities of this plant were measured in the present study.

During the pathogenesis of AMD, A2E produced from photoreceptor cells accumulates in the retinal pigment epithelium (RPE) of the retina and gradually transfers into oxidized forms (A2E-ox and A2E-2ox) on exposure to BL [41,42]. ROS produced from oxidized A2E induces cumulative damage through apoptosis, oxidative stress, and inflammation in the RPE [43]. Therefore, ARPE-19 cells are chosen as a representative RPE in most AMD studies [41]. The cell models for AMD are successfully induced in A2E-laden ARPE-19 cells after BL exposure, and they show significant alterations on the cell viability, antioxidant defense mechanism, apoptosis, inflammatory response, and angiogenesis [31]. Various natural products and compounds have been proven to prevent damage to the RPE in the AMD model generated in the A2E-laden ARPE-19 cells after exposure to BL [41]. Among these, photo-oxidation-induced apoptosis and death in A2E + BL-treated ARPE-19 cells were significantly protected against with grape skin polyphenols [44], *Vaccinium uliginosum* L. extract [45], polyphenol-enriched *Vaccinium uliginosum* [46], *Curcuma longa* L. extracts [47], *Arctium lappa* L. [48], *Prunella vulgaris var* L. [49], *Solanum melongena* L. [29], *Centella asiatica* [50], and *Dipterocarpus tuberculatus* [31]. Also, similar pretreatment effects were detected in the same cell model after treatment with single compounds, such as procyanidins B2 [51] and norbixin [52]. Furthermore, the attenuation of A2E accumulation, inhibition of ROS production, and activation of the antioxidant defense system in A2E-landen ARPE-19 cells after BL exposure were induced by the treatment with polyphenol-enriched *Vaccinium uliginosum* [46], *Arctium lappa* L. [48], *Prunella vulgaris var* L. [49], *Solanum melongena* L. [29], *Centella asiatica* [50], and *Dipterocarpus tuberculatus* [31]. But, the suppression of inflammatory responses was only induced by some natural products including *Prunella vulgaris var* L. [49], *Solanum melongena* L. [29], and *Dipterocarpus tuberculatus* [31] in A2E + BL-treated ARPE-19 cells. In the present study, we analyzed the suppression of intracellular free radical production, activation of the antioxidant defense system, and inhibition of the inflammatory response in A2E-laden ARPE-19 cells after exposure to BL to investigate the therapeutic effects and the mechanism of action of the MEE in AMD. Most of the results of the present study are consistent with previous studies, which showed the therapeutic effects and mechanism of action of several natural products in A2E + BL-exposed ARPE-19 cells. Therefore, our findings provide a scientific basis for novel natural products that have a therapeutic efficacy in AMD. Furthermore, the improvement effects of MEE on the dysregulation of angiogenesis caused by the A2E + BL exposure is first investigated in the present study. But, there were some difference in their effects. Especially, LMEE had no effect on the MMP-2 expression, while a significant decreasing effect was observed on only MMP-9 and VEGF expression. These differences are thought to be associated with the differential function of each protein during AMD although their expressions are tightly associated with angiogenesis [53]. Similar patterns on their expression after LMEE treatment are likely due to the positive feedback regulation between MMP-9 and VEGF expressions in RPE cells [54].

Until now, animal models based on the histological characteristics of AMD have been created in several animal species including mice, rats, rabbits, pigs, and non-human primates, although there is no model yet that has all the phenotypes of human AMD [55]. Specifically, rodent models for AMD have been widely used and applied in efficacy studies involving therapeutic drugs and in studies to evaluate causative mechanisms because of the low cost, the quick progression of disease, and the convenience of genetic manipulation [56]. Transgenic and knock-out mice exhibiting overexpression and deficiency of several genes involved in the complement factor pathway, chemokines, oxidative damage, and lipid/glucose metabolism exhibit some of the histological features that are detected in the eyes of AMD patients [57,58,59,60]. In addition to genetically modified models, BL exposure-induced animal models are commonly used for the efficacy studies of therapeutic products. These models showed the disorganization of the photoreceptor OS and inner segment (IS) after exposure to BL [31]. However, in this model, the treatment of A2E is not as necessary as that of ARPE-19 cells in order to induce a AMD condition because it forms as a byproduct of the visual cycle and accumulates with aging [4,6]. Also, the treatment with natural products resulted in a remarkable recovery of the altered histological structure of the retina. The polyphenol enriched *Vaccinium uliginosum* treatment improved the thickness of the ONL and the nuclei count of the retina in BALB/c mice exposed to BL [46], while similar protective effects were detected with the leaves extract of *Arctium lappa* L. and *Prunella vulgaris var* L [48,49]. Furthermore, the fundus damage and the degeneration of the retinal layer in BL-exposed BALB/c mice improved with the administration of *Solanum melongena* L. [29]. In another recent study, it was observed that retinal degeneration, including the decrease in OS, ONL, and INL thickness, was protected against by the extracts of *Dipterocarpus tuberculatus* [31]. In the present study, we verified the therapeutic effects of the MEE that were detected in A2E + BL-treated ARPE-19 cells using the BALB/c model with BL-induced retinal degeneration. The thicknesses of the whole retina, OS, ONL, and INL were remarkably increased in the BL-induced AMD model after the administration of the MEE. The results of the present study were very similar to the results of previous studies that investigated the therapeutic effects of various natural products on the retinas of BL-exposed BALB/c mice. However, the lack of detailed histopathological analyses, including that of the ocular fundus and the number of RPE cells in MEE + BL-treated BALB/c mice, should be considered a drawback of our study. Furthermore, there are a significant difference in the decreasing rate of iNOS expression between the ARPE-19 cells model and BALB/c model for AMD. The Western blot analyses showed about a 36–73% decreasing rate of iNOS expression levels in ARPE-19 cells after MEE treatment, but IHC staining analyses represented only a 9–27% decreasing rate of them in the BALB/c model after the same treatment. This difference between them is thought to be due to the detection accuracy of the analysis’s methods for iNOS proteins and the complexity of the cell composition of the experimental subject. However, our study has some limitations because it did not identify additional pathways involved in the protection effects of MEE. Therefore, additional studies are needed to identify a more comprehensive view of the global molecular changes using proteomic and transcriptomic analyses. Also, another limitation was that our study investigated only the short-term effects of MEE in AMD models. Additional studies are needed to assess chronic administration and its impact over time could improve the findings’ applicability after the observation of MEE effects for a longer period of time.

## 4. Materials and Methods

### 4.1. Preparation of MEE

The lyophilized sample of the MEE (FBM 223-005) was provided by the International Biological Material Research Center (IBMRC) of the Korea Research Institute of Bioscience and Biotechnology (KRIBB, Daejeon, Republic of Korea). First, the MEE was collected from a mixture of ground dried powder of *Euphorbia heterophylla* L. stems and leaves and methanol solution (1:10 ratio), as described in a previous study [31]. Prof. Young-Whan Choi, Department of Horticultural Bioscience, Pusan National University (Miryang, Republic of Korea), confirmed the samples to be *Euphorbia heterophylla* L. The mixture was firstly sonicated for 15 min and incubated at room temperature for 2h. Then, these steps were repeatedly conducted 10 times per day for 3 days. After collecting the extracted solution, the final MEE samples were prepared through a filtration process using a filter with 0.4 µm pore size and a lyophilization process using a rotary evaporator (*n* = 1000 SWD, EYELA, Bohemia, NY, USA) and a speed vacuum concentrator (Modulspin 40, Biotron Co., Marysville, WA, USA). Finally, the final samples were deposited as voucher specimens of *Euphorbia heterophylla* L. (WPC-23-001) at the functional materials bank of the Wellbeing RIS Center, Pusan National University (PNU). They were dissolved in dimethyl sulfoxide solution (DMSO; D1370.1000, Duchefa Biochemie, Haarlem, The Netherlands) at an appropriate concentration before being used to spontaneously treat ARPE-19 cells or BALB/c mice with AMD phenotypes.

### 4.2. Identification of Bioactive Compounds in MEE

The bioactive compounds in the MEE were analyzed using ultra-high-performance liquid chromatography–mass spectrometry (UHPLC-MS), as described in previous studies [61]. Liquid chromatography using a Water T3 column (2.1 × 100 mm, 1.8 µm) (Waters, Milford, MA, USA) was carried out. Mass spectrometric (MS) detection in the positive mode ([M + H]^+^ ions) was conducted using an Agilent 1290 Infinity HPLC system (Agilent Technologies, Waldbronn, Germany). The parameters for MS were selected as follows: gas temp, 300 °C; gas flow, 9 L/min; nebulizer pressure, 45 psig; VCap, 4000 V; and fragmentor voltage, 175 V. The MS spectrum of the bioactive compounds in the MEE showed a fragmentation pattern consistent with the MS data from the MassBank mass spectral database (http://massbank.jp/, accessed on 19 August 2024). Finally, the ratios of peak area were calculated from the observed peak areas in the total running time.

### 4.3. Free Radical Scavenging Activity MEE

The scavenging activity of MEE for the DPPH and ABTS radicals was measured using a previously described method [62]. To analyze the DPPH radical scavenging activity, a lyophilized sample of the MEE was dissolved in a 50% ethanol solution (100 µL) to obtain 12 solutions with varying concentrations of the MEE (1 to 125 µg/mL), which were then mixed with 100 µL of 0.1 mM DPPH (D9132, Sigma-Aldrich Co., St. Louis, MO, USA) in a 95% ethanol solution, or with 100 µL 95% ethanol solution (control), followed by incubation at room temperature for 30 min. A VersaMax^TM^ microplate reader (Molecular Devices, Sunnyvale, CA, USA) was used to determine the absorbance of the reaction mixture at 517 nm. The DPPH radical scavenging activity of the MEE was represented as the percent decrease in absorbance, relative to the control.

To analyze the ABTS radical scavenging activity, eleven different dosages of the MEE (1 to 1000 μg/mL, 25 μL) were mixed with the ABTS working solution (250 μL), and they were reacted at room temperature for 4 min. Finally, their absorbance was measured at 734 nm in an ultraviolet (UV)-visible (UV–VIS) spectrophotometer (Thermo Fisher Scientific Inc., Wilmington, DE, USA). The resultant data for the ABTS radical scavenging activity has been presented as ascorbic acid equivalent (A5960, Sigma-Aldrich Co.), which was used as a standard.

To measure the NO radicals scavenging activity, a total 100 µL of MEE solution with varying concentrations of the MEE (1 to 1000 µg/mL) was mixed with 400 µL of 10 mM sodium nitroprusside, and their reaction was induced at room temperature for 2.5 h. Then, this mixture was mixed with Griess reagent (200 µL) and incubated at room temperature for 30 min. Finally, the absorbance of reaction mixture was analyzed at 540 nm using a VersaMax^TM^ plate reader (Molecular Devices).

The scavenging activity of MEE against DPPH, ABTS, and NO radicals was represented as the IC_50_ value. This value indicates the MEE concentration that exerts a 50% decrease in the three radical scavenging activity. But, the maximum concentration of MEE was limited to the concentration at which each radical was 100% inhibited.

### 4.4. Synthesis and Purification of A2E

A2E for the induction of AMD into ARPE-19 cells was prepared, as described in a previous study [63]. Briefly, all-trans-retinal (100 mg, 352 μmol, R2500, Sigma-Aldrich Co.), ethanolamine (9.5 mg, 155 μmol, 15014, Sigma Aldrich Co.) in ethanol (3 mL), and acetic acid (9.3 µL, 155 μmol, 31010S0350, Junsei Chemical Co., Ltd., Tokyo, Japan) were mixed for 2 min and incubated at room temperature for 48 h under dark conditions. Then, its concentration was conducted at 20 °C using a nitrogen evaporator (Biotage, TurboVap-LV, Charlotte, NC, USA). A2E that was successfully synthesized was separated and purified using silica gel and HPLC with Sep-Pak C18 cartridge (WAT023635, Waters) using a step gradient elution with 5:95 methyl alcohol/dichloromethane (MeOH/CH_2_Cl_2_), 5:95 MeOH/CH_2_Cl_2_, and 8:92:0.001 MeOH/CH_2_Cl_2_/Trifluoroacetic acid (TFA). Additionally, the fractions were further purified using a multiple preparative HPLC (LC-forte/R, YMC Co., Kyoto, Japan) with a YMC-Triart Prep C18 column (250 mm × 10.0 mm, 10 μm). Finally, the gradient HPLC method with a YMC-Triart C18 column (4.6 mm × 250 mm, 5 µm) were performed to verify the purity of the A2E.

### 4.5. In Vitro Study

#### 4.5.1. Cell Culture and Cell Viabilities Assay

ARPE-19 cells, derived from the normal eyes of a 19-year-old male, were provided from the American Type Culture Collection (ATCC), (Manassas, VA, USA) and cultured in a 5% CO_2_ and 95% humidified atmosphere at 37 °C in Dulbecco’s Modified Eagle’s Medium (DMEM, LM001-05, Welgene, Gyeongsan-si, Republic of Korea) containing 10% fetal bovine serum (FBS), 2 mM glutamine, 100 U/mL of penicillin, and 100 μg/mL streptomycin.

The optimal concentrations of the MEE as well as A2E at specific BL irradiation levels were determined as described in a previous study [31]. Based on the preliminary results and treatment schedule (Supplementary Figure S2), the non-toxic concentration of the MEE was determined as 25, 50, 100, 200, and 400 μg/mL in the ARPE-19 cells (Supplementary Figure S3). Also, the optimal concentration of A2E was determined as 20 μM during the BL irradiation (430 nm, 6000 lux) (SL-S2500, S tech LED, Gyeonggido, Republic of Korea) for 10 min (Supplementary Figure S4). Finally, the optimal treatment concentration of the MEE under the optimal A2E concentration and BL irradiation was determined as 50, 100, and 200 μg/mL in the ARPE-19 cells (Supplementary Figure S5).

To examine the protective effects of MEE on the AMD, induced by BL in A2E-laden ARPE-19 cells, the cells were briefly classified into two groups: No group (non-treated group) and the A2E + BL-treated group. The latter group was further divided into the four groups: Vehicle (DMSO)-treated group (Vehicle + A2E + BL-treated group), low-dose (50 μg/mL) MEE-treated group (LMEE + A2E + BL-treated group), medium-dose (100 μg/mL) MEE-treated group (MMEE + A2E + BL-treated group) and high-dose (200 μg/mL) MEE-treated group (HMEE + A2E + BL-treated group). ARPE-19 cells were cultured to 70–80% confluence and pretreated with 50, 100, and 200 μg/mL of the MEE or DMSO of the same volume to have a final concentration of 1% for 24 h. The cells of each group were further treated with 20 μM of A2E for 24 h. Then, the MEE and A2E-treated cells were irradiated by BL (430 nm, 6000 lux) for 10 min. The viability of the cells was measured by a 3-[4,5-dimethylthiazol-2-yl]-2,5 diphenyl tetrazolium bromide (MTT) assay as described in previous studies after incubation for 24 h [31] (Supplementary Figure S2). Finally, the cells of each groups were used for molecular analyses including fluorescence-activated cell sorting (FACS), the Western blot test, and real-time-quantitative polymerase chain reaction (RT-qPCR) analyses.

#### 4.5.2. Analysis of the Apoptotic Cell Population

The distribution of the apoptotic cell population was analyzed using a Muse^TM^ Annexin V and Dead Cell Kit (MCH100105, Millipore Co., Billerica, MA, USA), based on the manufacturer’s protocol [64]. After harvesting the ARPE-19 cells treated with the MEE, A2E, and BL, they (1 × 10^4^ cells/mL) were stained with the reaction reagent of the Muse^TM^ Annexin V and Dead Cell Kit (Millipore Co.) for 20 min at room temperature. These cells were analyzed using the Muse^TM^ Cell Analyzer (Millipore Co.), and their distribution was presented as the population of live and apoptotic cells. After gating based on cell size, the cell population was distinguished into four different groups: non-apoptotic cells [Annexin V (−) and 7-AAD (−)], early apoptotic cells [Annexin V (+) and 7-AAD (−)], late apoptotic cells [Annexin V (+) and 7-AAD (+)], and mostly nuclear debris [Annexin V (+) and 7-AAD (+)].

#### 4.5.3. Intracellular ROS

The intracellular ROS levels were measured by observing the ARPE-19 cells stained with 2′,7′-dichlorodihydrofluorescein diacetate (DCFH-DA; D6883, Sigma-Aldrich Co.) under a fluorescent microscope, as described in a previous study [65]. After the final treatment with the MEE, A2E, and BL, these cells were further incubated with 10 µM 2′-7′-dichlorofluorescein diacetate (DCF-DA) for 30 min at 37 °C. After washing with 1× phosphate-buffered saline (PBS) twice, all positive cells with green fluorescence were detected using a fluorescent microscope (Evos m5000, Thermo Fisher Scientific Inc.) at 400× magnification, and counted in counted in two fields of view (67,500 mm^2^) in each well. Then, their levels were represented as relative value for the control group.

#### 4.5.4. Analysis of NO

The NO concentration and the level of nitrite in the ARPE-19 cells were analyzed using the Griess reagent (G7921, Invitrogen Co, Waltham, MA, USA), as outlined in a previous study [66]. After collecting the culture supernatants, they (100 μL) were mixed with the modified Griess reagent (Invitrogen Co.) (100 μL) and incubated at room temperature for 5 min. Then, the absorbance of each reaction was determined at 540 nm using a Versamax^TM^ microplate reader (Molecular Devices). Finally, the NO concentration of each sample was quantified using a standard curve with sodium nitrite.

#### 4.5.5. Analysis of SOD Activity

The SOD activity in MEE + A2E + BL-treated ARPE-19 cells was determined using a SOD assay kit (S311, Dojindo Molecular Technologies Inc., Rockville, MD, USA) according to the manufacturer’s protocol and previous study [67,68]. After collecting the total lysate of the ARPE-19 cells using repetitive freezing and thawing, they were diluted with 1× PBS in seven ratios (1/1, 1/5, 1/52, 1/53, 1/54, 1/55, and 1/56) in individual wells. This solution was thoroughly mixed with 200 μL water-soluble tetrazolium salt-1 (WST-1) working solution and 20 μL enzyme working solution. The enzyme reaction of this mixture was promoted by incubation at 37 °C for 20 min, after which the absorbance of each well was measured at 450 nm using a Versamax^TM^ microplate reader (Molecular Devices). Finally, the SOD activities were calculated using the following equation: SOD activity (inhibition rate %) = [(A blank 1-A blank 3)-(A sample-A blank 2)]/(A blank1-A blank3) × 100(1), where A blanks 1, 2, and 3 indicate the absorbance of blanks 1, 2, and 3, respectively, and ‘A sample’ is the sample absorbance.

#### 4.5.6. Western Blot Analysis

The total cellular proteins were obtained from the ARPE-19 cells of subset groups using the Pro-Prep Protein Extraction Solution (17081, Intron Biotechnology Inc., Seongnam, Republic of Korea), based on the manufacturer’s protocol [69]. After centrifugation at 13,000 rpm for 5 min, the protein concentrations of the supernatant were determined using a SMART^TM^ Bicinchoninic Acid (BCA) Protein Assay Kit (23225, Thermo Fisher Scientific Inc.). They were separated by 4–20% sodium dodecyl sulfate-polyacrylamide gel electrophoresis (SDS-PAGE) for 2 h, followed by transfer to nitrocellulose membranes at 40 V for 2 h. The membranes were then incubated overnight at 4 °C with the following primary antibodies: anti- Bax (2772s, Cell signaling Technology Inc., Danvers, MA, USA), anti-Bcl-2 (PA5-20068, Invitrogen Co.), anti-Cas-3 (9662s, Cell signaling Technology Inc.), anti-Nrf2 (ab137550, Abcam, Cambridge, UK), anti-phospho (p)-Nrf2 (PA5-67520, Invitrogen Co.), anti-SOD (ab13498, Abcam), anti-MMP-2 (40994s, Cell signaling Technology Inc.), anti-MMP-9 (2270s, Cell signaling Technology Inc.), anti-VEGF (ab46154, Abcam), anti-iNOS (13120s, Cell Signaling Technology Inc., Denvers, MA, USA), anti- COX-2 (12282s, Cell Signaling Technology Inc.), anti-ASC (67824s, Cell Signaling Technology Inc.), anti-Cas-1 (24232s, Cell Signaling Technology Inc.), anti-NLRP3 (15101s, Cell Signaling Technology Inc.), and anti-β-actin antibody (4967s, Cell signaling Technology Inc.). Membranes were then washed with washing buffer (137 mM sodium chloride (NaCl), 2.7 mM potassium chloride (KCl), 10 mM disodium phosphate (Na_2_HPO_4_), and 0.05% Tween 20), followed by incubation with 1:2000 diluted horseradish peroxidase (HRP)-conjugated goat anti-rabbit immunoglobulin G (IgG) (G21234, Invitrogen Co.), at room temperature for 1 h. Blots were developed using the EZ-Western Lumi Femto Kit (DG-WF100, Dogen, Seoul, Korea). Chemiluminescence signals were analyzed using the Fusion Solo-2 imaging system (Vilber, San Leandro, Collégien, France). Finally, the intensity of each protein band was determined using the AlphaView Program, version 3.2.2 (Cell Biosciences Inc., Santa Clara, CA, USA) and the relative levels of this protein was determined based on the intensity of actin as endogenous housekeeping protein.

#### 4.5.7. Real-Time Quantitative PCR Analysis

The total RNA was purified from the ARPE-19 cells of the subset groups using TRIzol reagent (FATRR001, Favorgen Biotech, Ping-Tung, Taiwan), based on the manufacturer’s protocol [70]. The complementary DNA (cDNA) was synthesized from total RNA (20 μg) using the Superscript II reverse transcriptase (18064-014, Invitrogen Co.), and subsequently quantitative PCR (qPCR) was performed using the cDNA template (2 μL) and 2× Power SYBR Green (10 μL; QPK-201, Toyobo Life Science, Osaka, Japan) containing specific primers as follows: TNF-α, sense 5′-CCTGT AGCCC ACGTC GTAGC-3′, and anti-sense 5′-TTGAC CTCAG CGCTG ACTTG-3′, IL-6, sense 5′-TTGGG ACTGA TGTTG TTGAC A-3′, and anti-sense 5′-TCATC GCTGT TGATA CAATC AGA-3′, IL-1β, sense 5′-CAGTT CTGCC ATTGA CCAT-3′, and anti-sense 5′-TCTCA CTGAA ACTCA GCCGT-3′; NF-κB, sense 5′-GTAAC AGCAG GACCC AAGGA-3′, and anti-sense 5′-AGCCC CTAAT ACACG CCTCT-3′; VEGF, sense 5′-GGAGGCGCAGCGGTTAG-3′, and anti-sense 5′-AACCCGGATCAATGAATATCAAA-3′; β-actin, sense 5′-TGGAA TCCTG TGGCA TCCAT GAAAC-3′, and anti-sense 5′-TAAAA CGCAG CTCAG TAACA GTCCG-3′. The qPCR was performed over 40 cycles consisting of denaturation (95 °C for 15 s), annealing (70 °C for 60 s), and extension (70 °C for 60 s). The transcription levels of TNF-α, IL-6, IL-1β, NF-κB, and VEGF genes were determined relative to that of the housekeeping gene β-actin, based on a comparison of the Ct values at a constant fluorescence intensity [71].

### 4.6. In Vivo Study

#### 4.6.1. Experimental Design for Animal Study

The Pusan National University (PNU)–Institutional Animal Care and Use Committee (IACUC) reviewed and approved the protocol for the AMD animal model study (Approval no. PNU-2022-0249). All mice were housed at the PNU-Laboratory Animal Resources Center (LARC) accredited by the Korean Food and Drug Administration (KFDA) (unit 000231) and the Association for Assessment and Accreditation of Laboratory Animal Care International (AAALAC International) (unit 001525). Male BALB/c mice (7 weeks old) originated from Taconic Co. (Germantwon, NY, USA) and were purchased from the Samtako BioKorea Co., Ltd. (Osan, Republic of Korea). The mice were bred under specific pathogen-free conditions (SPF) (50 ± 10% of relative humidity and 23 ± 2 °C of temperature) under a strict light/dark cycle.

Briefly, the 7-week-old BALB/c mice (thirty males with an average weight of 20.29 g) were assigned to either a non-irradiated group (non-treated group, *n* = 7) or a BL irradiated group (*n* = 21). The BL irradiated group was further divided into the Vehicle-treated group (1× PBS, Vehicle + BL-treated group, *n* = 7), low-concentration MEE-treated group (100 mg/kg, LMEE + BL-treated group, *n* = 7), or high-concentration MEE-treated group (200 mg/kg, HMEE + BL-treated group, *n* = 7). After adapting to the dark conditions for three days, all the mice in the BL irradiated groups were orally administrated with 100 mg/kg, 200 mg/kg of MEE, or the same volume of 1× PBS once a day for 4 days in dark cages. Subsequently, they were exposed to BL for 2 h and then bred for 24 h under dark conditions. Finally, all the BALB/cKorl syngeneic mice were euthanized by skilled researchers using CO_2_ gas with a minimum purity of 99.0% based on the American Veterinary Medical Association (AVMA) Guidelines for the Euthanasia of Animals. The death of the mice was confirmed by ascertaining cardiac and respiratory arrest, or dilated pupils and a fixed body. The eye samples were then collected from all euthanized BALB/c mice.

#### 4.6.2. Histopathological Analysis

After collection of the eye tissues from the BL-exposed BALB/c mice, these tissues were fixed in 10% neutral buffered formaldehyde (pH 6.8), dehydrated in an alcohol dilution series (70 to 100%), and embedded in paraffin wax. The tissues were cut into sections of 4 µm thickness, and subsequently deparaffinized and rehydrated using xylene solution (8587-4410, DaeJung Chemicals, Siheung, Republic of Korea) and an alcohol dilution series (100 to 70%). After removing the residues with distilled water, the eye tissue sections were stained with hematoxylin and eosin (H&E; Sigma-Aldrich Co.), and the histopathological changes in eye tissues were analyzed using the Leica Application Suite (LAS, Leica Microsystems). The thickness of the whole retina, OS, the ONL, INL, and IPL of the mounted eye tissue were measured using the Leica Application Suite (Leica Microsystems, Switzerland). Considering the surface irregularities of each layer, three different points including thickest point, thinnest point, and medium thickness point were measured and presented as their mean values.

#### 4.6.3. Immunohistochemical Staining Analysis

The tissue distribution of the COX-2, and iNOS proteins was detected using immunohistochemical (IHC) staining analysis as described in a previous study [72,73]. The deparaffinized and rehydrated tissue sections were pretreated with a blocking buffer for 30 min. They were bounded with anti-COX-2 (Cell Signaling Technology Inc.) and anti-iNOS (PA3-030A, Thermo Fisher Scientific Inc.) primary antibodies diluted 1:200. After washing in a 1× PBS solution, these slides were bounded with goat HRP conjugated anti-rabbit IgG (1:200, Invitrogen Co.) for 45 min. Finally, the distribution of each protein in the retina was detected as a dark brown color using the stable 3,3′-diaminobenzidine (DAB) reaction (D39-18, GBI Labs, Bothell, WA, USA) and the color density for iNOS and COX-2 expression was quantified using the Image J program 1.52a (NIH, Bethesda, ML, USA) together with Image-Color-Split Channels and the Analysis-Tools-ROI Manager.

### 4.7. Statistical Analysis

Briefly, the normality of distribution of all data were checked by K-S test. Statistical analyses were performed using the SPSS release 10.10 for Windows (IBM SPSS, SPSS Inc., Chicago, IL, USA). The significance of the intergroup differences was determined by a one-way analysis of variance, followed by Tukey’s post hoc test for multiple comparisons. Results are presented as means ± SDs, and statistical significance was accepted for *p* values < 0.05.

## 5. Conclusions

In the present study, we attempted to identify novel natural products with a high potential for AMD treatment using an in vitro and in vivo model. To achieve this, the MEE was selected based on its antioxidant activity, and the antioxidant defense mechanism, anti-apoptosis pathway, anti-angiogenesis effects, and anti-inflammatory response of the MEE were analyzed in A2E-laden ARPE-19 cells after exposure to BL; the retinas of BL-exposed BALB/c mice were also analyzed. The suppression of intracellular oxidative stress and activation of antioxidant defense mechanism were detected in A2E + BL-treated ARPE-19 cells. Also, the MEE treatment inhibited the inflammatory response, apoptosis activation, and upregulation of neovascular protein induced by A2E + BL treatment. Furthermore, significant enhancement effects of MEE on the retinal thickness were detected in BALB/c mice with BL-induced retinal degeneration. Therefore, these results suggest that the MEE, with its high antioxidative activity, protects against BL-induced retinal degeneration through the regulation of the antioxidative system, inflammatory response, apoptosis, and neo-vascularization in the AMD mouse model.

## Figures and Tables

**Table 1:** Chemical properties and relative contents of ten compounds in MEE.

No.	Compound Name	Molecular Formula	Molecular Weight (g/mol)	Time (min)	Expected *m*/*z*	Polarity	Observed *m*/*z*	Relative Content (%)
1	Ellagic acid	C_14_H_6_O_8_	302.19	0.92	303.2121	[M + H]^+^	303.1991	2.81
2	β-sitosterol	C_29_H_50_O	414.71	10.62	415.3932	[M + H]^+^	415.1879	0.03
3	Quercetin	C_15_H_10_O_7_	302.24	11.67	303.0499	[M + H]^+^	303.0506	8.46
4	Quercetrin	C_21_H_20_O_11_	448.38	12.56	449.1078	[M + H]^+^	449.1088	7.13
5	Kaempferol	C_15_H_10_O_6_	286.23	13.36	287.055	[M + H]^+^	287.0548	5.79
6	Octanoic acid	C_8_H_16_O_2_	144.21	18.10	145.1223	[M + H]^+^	144.9825	0.13
7	Palmitoleamide	C_16_H_31_NO	253.42	28.20	254.2478	[M + H]^+^	254.2484	11.15
8	Linoleamide	C_18_H_33_NO	279.50	29.55	280.2635	[M + H]^+^	280.264	14.20
9	Linoleic acid	C_18_H_30_O_2_	278.43	31.12	279.2319	[M + H]^+^	279.2359	0.06
10	Oleamide	C_18_H_35_NO	281.48	32.08	282.2791	[M + H]^+^	282.2796	50.24

Abbreviations: M, molecular compound; H, additional proton.

**Table 2:** Total cell number of each stage in apoptosis.

Cell State	No	A2E + BL			
		Vehicle	LMEE	MMEE	HMEE
Live cells	1.5 × 10^6^ ± 8.71 × 10^3^	2.4 × 10^5^ ± 3.61 × 10^2^	3.03 × 10^6^ ± 4.37 × 10^3^	2.9 × 10^6^ ± 3.08 × 10^3^	2.12 × 10^6^ ± 1.39 × 10^3^
Early apoptotic cells	1.51 × 10^3^ ± 2.64 × 10 ^2^	3.31 × 10^4^ ± 64.6	1.1 × 10^4^ ± 3.89 × 10^2^	7.05 × 10^3^ ± 2.92 × 10^2^	2.97 × 10^3^ ± 30.7
Late apoptotic cells	1.04 ± 2.07	6.88 × 10^2^ ± 2.12	2.06 × 10^2^ ± 5.93	9.07 × 10 ± 5.6	1.5 ± 0.43
Dead cells	4.35 × 10^−2^ ± 2.24 × 10^−2^	2.01 × 10 ± 2.06	15.9 ± 2.24	37.7 ± 1.13	0.27 ± 0.14
Total apoptotic cells	1.59 × 10^3^ ± 2.66 × 10^2^	4.34 × 10^4^ ± 69.6	1.43 × 10^4^ ± 2.99 × 10^2^	8.71 × 10^3^ ± 2.28 × 10^2^	3.1 × 10^3^ ± 36.2

Abbreviations: No, No treated group; LMEE, Low concentration of methanol extracts of *Euphorbia heterophylla* L.; MMEE, Medium concentration of methanol extracts of *Euphorbia heterophylla* L.; HMEE, High concentration of methanol extracts of *Euphorbia heterophylla* L.; A2E, N-retinylidene-N-retinylethanolamine; BL, blue light.

**Table 3:** Thickness of each layer in the retina.

Layer	No		BL	
		Vehicle	LMEE	HMEE
OS (μm)	10.89 ± 0.23	6.14 ± 0.22 *	8.02 ± 0.44 *^,#^	8.29 ± 0.47 *^,#^
ONL (μm)	40.89 ± 2.43	38.85 ± 3.41	36.77 ± 1.12 *^,#^	45.01 ± 4.38 *^,#^
INL (μm)	16.83 ± 1.87	11.91 ± 1.15 *	21.47 ± 0.92 *^,#^	13.83 ± 0.85 *
IPL (μm)	10.83 ± 0.79	10.56 ± 1.21	11.176 ± 0.81	11.59 ± 0.93
Whole retina (μm)	79.44 ± 5.32	67.46 ± 5.99 *	77.436 ± 3.29 ^#^	78.72 ± 6.63 ^#^

Abbreviations: OS, outer segment; ONL, outer nuclear layer; INL, inner nuclear layer; IPL, inner plexiform layer; LMEE, Low concentration of methanol extracts of *Euphorbia heterophylla* L.; HMEE, High concentration of methanol extracts of *Euphorbia heterophylla* L. Data are reported as the mean ± SD. * *p* < 0.05 vs. No group. # *p* < 0.05 vs. Vehicle + A2E + BL-treated group.

## Data Availability

Data are contained within the article and Supplementary Materials.

## Supplementary Materials

The following supporting information can be downloaded at https://www.mdpi.com/article/10.3390/ph17091193/s1, Figure S1: Chemical structure of ten active compounds including ellagic acid, β-sitosterol, quercetin, quercitrin, kaempferol, octanoic acid, palmitoleamide, linoleamide, linolenic acid, and oleamide in the MEE; Figure S2: Treatment schedule for each experiment; Figure S3: Determination of the optimal concentration of the MEE; Figure S4: Determination of the optimal concentration for A2E treatment under BL exposure; Figure S5: Determination of the optimal concentration of MEE under the treatment of optimal A2E and BL conditions.
