# Supplementary material for: Novel Therapeutic Effects of Euphorbia heterophylla L. Methanol Extracts in Macular Degeneration Caused by Blue Light in A2E-Laden ARPE-19 Cells and Retina of BALB/c Mice
Source: Pharmaceuticals (Basel). 2024 Sep 10;17(9):1193. doi: 10.3390/ph17091193 (PMC11435363; doi:10.3390/ph17091193)
Supplement: Supplementary file 1 [file pharmaceuticals-17-01193-s001.zip › pharmaceuticals-3190918-supplementary.pdf]

Supplementary Figures

Novel therapeutic effects of *Euphorbia heterophylla* L. in blue light-induced macular degeneration in A2E-laden ARPE-19 cells and retina of BALB/c mice

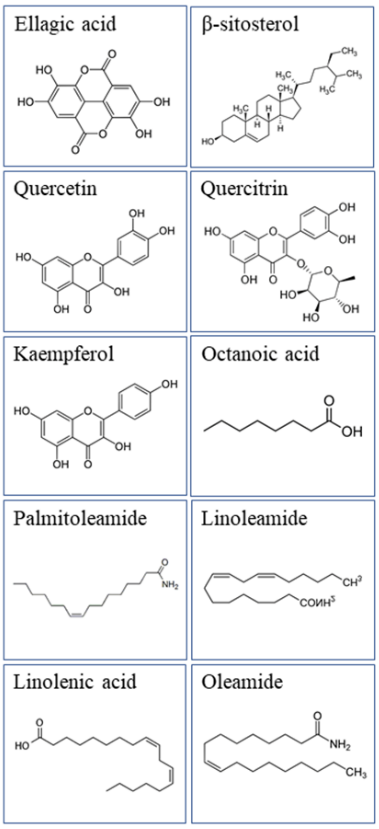

Supplementary Figure S1. Chemical structure of ten active compounds including ellagic acid, β-sitosterol, quercetin, quercitrin, kaempferol, octanoic acid, palmitoleamide, linoleamide, linolenic acid, and oleamide in the MEE. Abbreviations: MEE, Methanol extracts of *Euphorbia heterophylla* L.

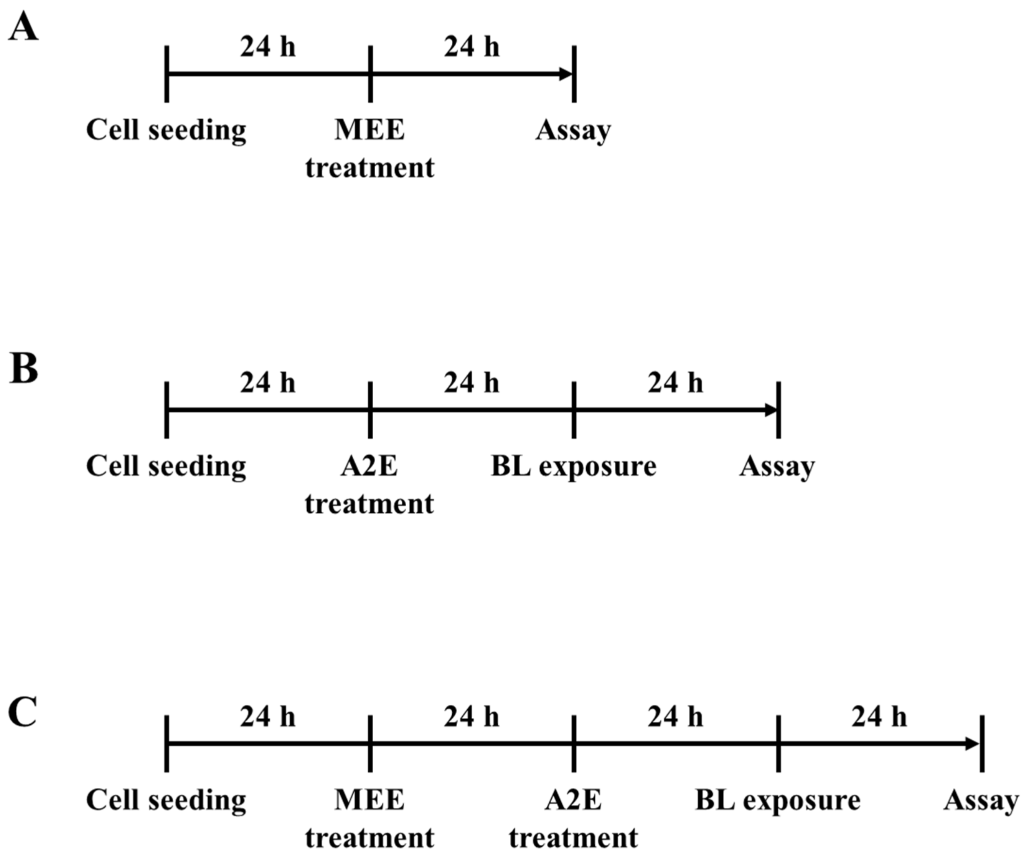

Supplementary Figure S2. Treatment schedule for each experiment. (A) Experimental schedule for determination of the optimal concentration of MEE. (B) Experimental schedule for determination of the optimal concentration of A2E treatment under BL exposure. (C) Experimental schedule for evaluating the efficacy of MEE under the treatment of optimal A2E and BL conditions. Abbreviations: MEE, Methanol extracts of *Euphorbia heterophylla* L.; A2E, N-retinylidene-N-retinylethanolamine; BL, Blue light

A

B

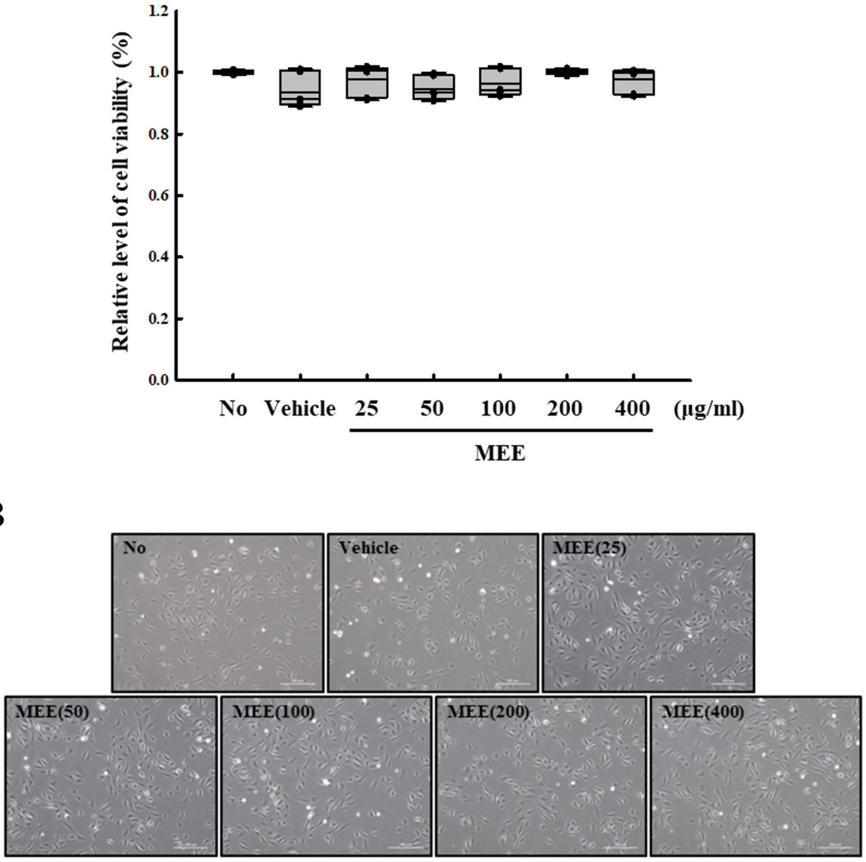

Supplementary Figure S3. Determination of the optimal concentration of the MEE. **(A)** Relative level of cell viability. **(B)** Morphology of ARPE-19 cells. After incubation of ARPE-19 cells with the Vehicle, 25, 50, 100, 200, and 400 µg/mL MEE for 24 h, the morphological changes in the cells were observed under a microscope at 400× magnification. After pretreatment with three different dosages of the MEE for 24 h, their viability was analyzed using the MTT assay. The MTT assays were performed from two to three wells per group, and the optical density was measured twice for each well. Data are reported as the mean ± SD. **p*<0.05 vs. non-treated group. Abbreviations: MTT, 3-(4,5-dimethylthiazol-2-yl)-2,5-diphenyltetrazolium bromide; MEE, Methanol extracts of *Euphorbia heterophylla* L; ARPE, arising retinal pigment epithelia.

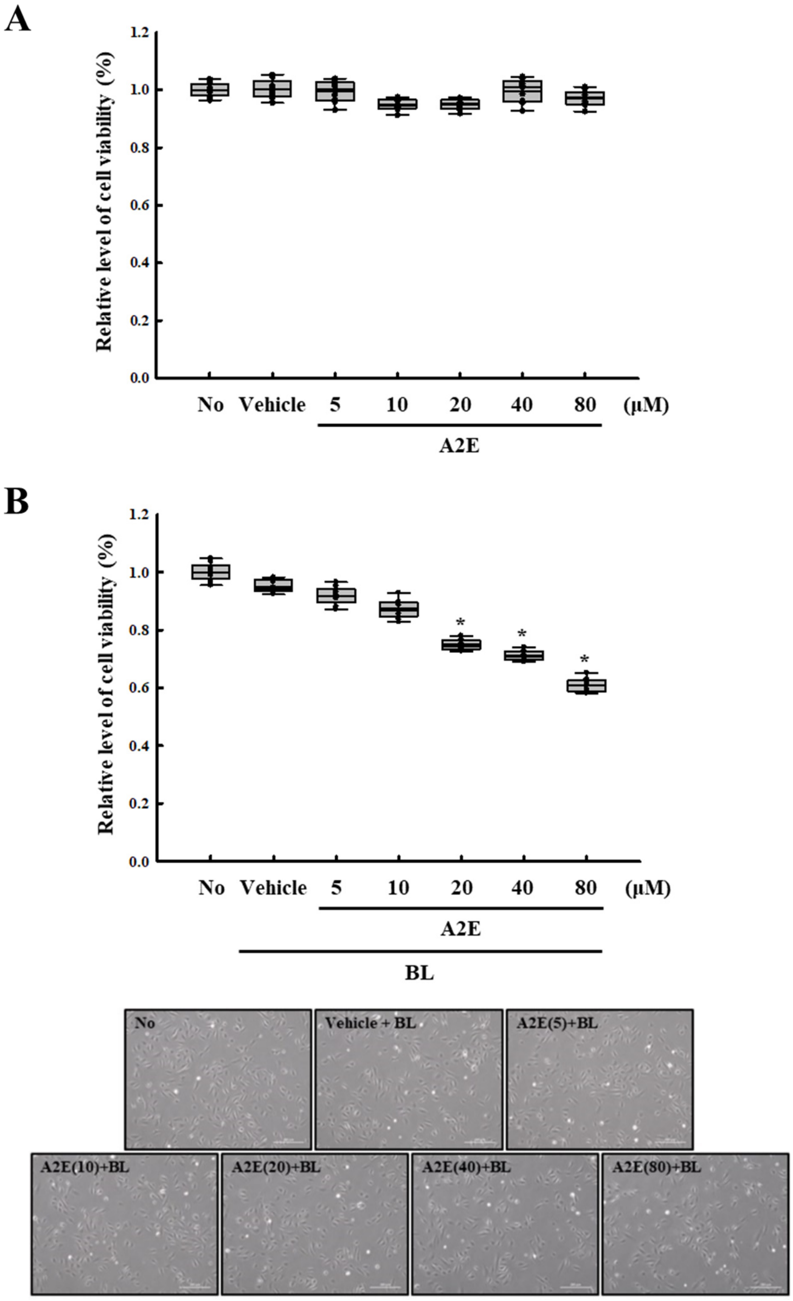

Supplementary Figure S4. Determination of the optimal concentration for A2E treatment under BL exposure. **(A)** Relative level of cell viability. **(B)** Relative level of cell viability and morphology of ARPE-19 cells. After incubation of the ARPE-19 cells with the Vehicle, 5, 10, 20, 40, and 80 µM A2E for 24 h, they were exposed to BL (430 nm, 6000 Lux) for 10 min, the morphological changes of the cells were observed under a microscope at 400× magnification. The MTT assays were performed from two to three wells per group, and the optical density was measured twice for each well. Data are reported as the mean ± SD. *p<0.05 vs. non-treated group. Abbreviations: MTT, 3-(4,5-dimethylthiazol-2-yl)-2,5-diphenyltetrazolium bromide; MEE, Methanol extracts of *Euphorbia heterophylla* L.; A2E, N-retinylidene-N-retinylethanolamine; BL, Blue light; ARPE, arising retinal pigment epithelia.

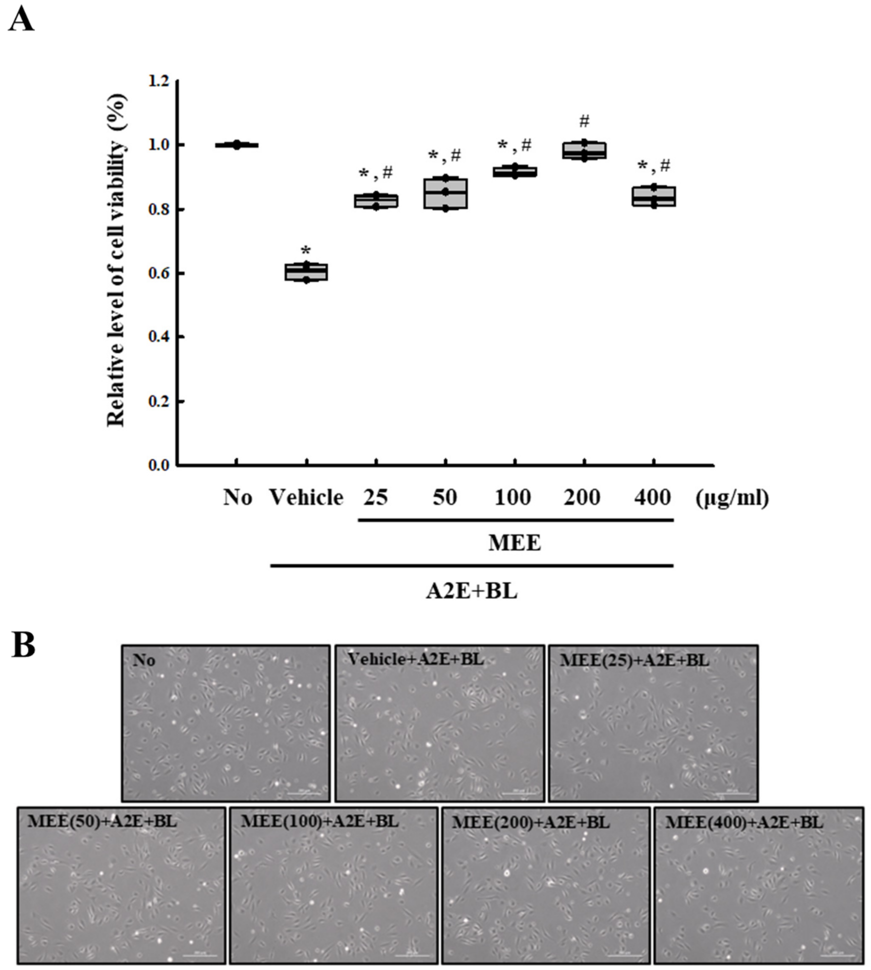

Supplementary Figure S5. Determination of the optimal concentration of MEE under the treatment of optimal A2E and BL conditions. **(A)** Relative level of cell viability. **(B)** Morphology of ARPE-19 cells. After incubation of ARPE-19 cells with Vehicle, 25, 50, 100, 200, and 400 μg/mL MEE for 24 h under the treatment of optimal A2E and BL conditions, the morphological changes of cells were observed under a microscope at 400× magnification, and their viability was analyzed using the MTT assay. The MTT assays were performed from two to three wells per group, and the optical density was measured twice for each well. Data are reported as the mean ± SD. **p*<0.05 vs. non-treated group. #*p*<0.05 vs. Vehicle+A2E+BL treated group. Abbreviations: MTT, 3-(4,5-dimethylthiazol-2-yl)-2,5-diphenyltetrazolium bromide; MEE, Methanol extracts of *Euphorbia heterophylla* L.; A2E, N-retinylidene-N-retinylethanolamine; BL, Blue light; ARPE, arising retinal pigment epithelia.
